# Optimizing Timing of Intraperitoneal Chemotherapy to Enhance Intravenous Carboplatin Concentration

**DOI:** 10.3390/cancers16162841

**Published:** 2024-08-14

**Authors:** Kohei Tamura, Natsuka Kimura, Hideyuki Ohzawa, Hideyo Miyato, Naohiro Sata, Takahiro Koyanagi, Yasushi Saga, Yuji Takei, Hiroyuki Fujiwara, Ryozo Nagai, Joji Kitayama, Kenichi Aizawa

**Affiliations:** 1 Department of Obstetrics and Gynecology, Jichi Medical University, Tochigi 329-0498, Japan; 2 Division of Clinical Pharmacology, Department of Pharmacology, Jichi Medical University, Tochigi 329-0498, Japan; 3 Department of Surgery, Jichi Medical University, Tochigi 329-0498, Japan; 4 Division of Translational Research, Clinical Research Center, Jichi Medical University Hospital, Tochigi 329-0498, Japan; 5 Jichi Medical University, Tochigi 329-0498, Japan; 6 Clinical Pharmacology Center, Jichi Medical University Hospital, Tochigi 329-0498, Japan

**Keywords:** gastric cancer, peritoneal metastases, intraperitoneal chemotherapy, intravenous chemotherapy, interval timing, paclitaxel, carboplatin

## Abstract

**Simple Summary:**

Intraperitoneal (IP) administration of Paclitaxel (PTX) is a logical approach for treating peritoneal metastases (PMs), as it allows direct drug infiltration into PMs, providing prolonged exposure at high concentrations with minimal systemic side effects. However, the optimal timing for combining intravenous (IV) and IP administration remains uncertain. This study demonstrated that IP administration of PTX resulted in higher concentrations of PTX in peritoneal tumors, as well as increased levels of intravenously administered Carboplatin (CBDCA) when given four days after PTX administration. These findings suggest that IP PTX may enhance the efficacy of subsequent systemic chemotherapy. Therefore, staggered and repeated systemic chemotherapy following IP PTX appears to be a highly effective strategy for managing PMs.

**Abstract:**

Despite advances in systemic chemotherapy, patients with gastric cancer (GC) and peritoneal metastases (PMs) continue to have poor prognoses. Intraperitoneal (IP) administration of Paclitaxel (PTX) combined with systemic chemotherapy shows promise in treating PMs from GC. However, methods of drug administration need to be optimized to maximize efficacy. In this study, we utilized a mouse model with PMs derived from a human GC cell line, administering PTX either IP or intravenously (IV), and Carboplatin (CBDCA) IV 0, 1, and 4 days after PTX administration. The PMs were resected 30 min later, and concentrations of PTX and CBDCA in resected tumors were measured using liquid chromatography–tandem mass spectrometry (LC-MS/MS). Results indicated that PTX concentrations were higher with IP administration than with IV administration, with significant differences observed on days 0 and 1. CBDCA concentrations 4 days post-IP PTX administration were higher than with simultaneous IV PTX administration. These findings suggest that IP PTX administration enhances CBDCA concentration in peritoneal tumors. Therefore, sequential IV administration of anti-cancer drugs appears more effective than simultaneous administration with IP PTX, a strategy that may improve prognoses for patients with PMs.

## 1. Introduction

Gastric cancer (GC) generally has poor prognosis. Approximately 1,100,000 new cases and 770,000 deaths were registered worldwide in 2020, ranking GC as the fifth most diagnosed cancer and the third leading cause of cancer death [1]. Peritoneal metastases (PMs) from GC are associated with particularly dismal prognoses [2] because PMs cause various unpleasant symptoms and functional disorders due to large amounts of ascites and intestinal obstructions [3]. Furthermore, PMs are the main site of recurrence in patients with GC who underwent gastrectomy with curative intent [4,5,6]. Therefore, suppressing PMs is imperative to reduce unpleasant symptoms and prolong the survival of gastric cancer patients.

The current standard therapy for GC patients with PMs in Asia is intravenous (IV) and oral administration of Cisplatin and S-1 (tegafur-gimeracil-oteracil) [7,8]. However, so far, systemic chemotherapy alone or in combination with gastrectomy has failed to prolong the survival of GC patients with PMs, despite advances in anti-cancer drugs [9,10,11]. One reason that IV administration has little effect on PMs is the peritoneum–plasma barrier, which impedes drug delivery from the general circulation [12].

Considering anatomical features, Dedrick et al. first showed the effectiveness of intraperitoneal (IP) chemotherapy against PMs in 1978, and since then, many studies have demonstrated that drug concentrations in the peritoneal cavity are augmented by IP administration. IP administration has more direct effects on PMs and free intraperitoneal cancer cells, drug half-lives are longer, and infiltration of tumors is deeper than with systemic administration [13,14,15,16]. From a pharmacokinetic perspective, Paclitaxel (PTX) is suitable for IP chemotherapy because of its large particle size, hydrophobicity, and potent anti-tumor effects [17]. In fact, IP administration of PTX has proven highly effective for peritoneal lesions in various malignancies, such as ovarian [18,19] and gastric cancers [20]. In a phase III trial for GC, median survival times of the IP and IV PTX plus S-1 group tended to be longer than the IV Cisplatin plus S-1 group. However, adjusted for baseline ascites in the sensitivity analysis, overall survival was significantly better in the IP group, suggesting that IP PTX may be advantageous for gastric cancer treatment [21]. Many studies have demonstrated that the addition of nanoparticles or high-molecular-weight agents to prolong residence time in the abdominal cavity can increase IP PTX infiltration into tumors, suppressing PMs, and improving the clinical course of advanced-stage GC [22,23,24,25].

Although some studies demonstrated that a combination of IP and systemic chemotherapy is more effective against PMs [21,26], no studies have investigated mechanisms affecting concentrations of anti-cancer drugs administered with IV administration and interval timing between IP and IV administration, which encouraged us to explore concentrations of PTX and platinum-based drugs in tumors when IV Carboplatin (CBDCA) is administered after IP or IV PTX. We administered PTX either IP or IV and CBDCA IV, 0, 1, and 4 days after PTX administration in a mouse model of PMs. We then compared concentrations of PTX and CBDCA between the IP and IV PTX groups at the different times.

## 2. Materials and Methods

### 2.1. Cell Culture

MKN45 is a human gastric cancer cell line initially established from poorly differentiated gastric carcinoma tissue, and MKN45P, which has characteristics of highly metastatic capacity, was obtained from the University of Tokyo [27]. MKN45P was cultured in Dulbecco’s Modified Eagle’s Medium (DMEM) supplemented with 100 U/mL penicillin, 100 mg/mL streptomycin (Life Technologies, Grand Island, NY, USA), and 10% fetal bovine serum (FBS; Sigma, St. Louis, MO, USA). Cells were maintained with three passages in the same medium and used for experiments.

### 2.2. Drugs and Animals

PTX and CBDCA were obtained from Wako Pure Chemical (Osaka, Japan). PTX was dissolved in Cremophor EL:ethanol at a volume ratio of 1 to 1. It was adjusted to a concentration of 6 mg/mL and used after dilution with PBS to a final concentration of 400 µg/mL for IP administration, or 2 mg/mL for IV administration. A total of 1 mL of PTX solution was used for IP administration, and 200 µL for intravenous administration, resulting in a 400 µg PTX dose for mice in both groups. As a control, PTX vehicle was also used. CBDCA was dissolved in distilled water and 10× PBS to correct osmotic pressure to a concentration of 10 mg/mL, and 120 µL, i.e., 120 µg, of CBDCA was administered IV.

Specific-pathogen-free, four-week-old female BALB/c nude mice were purchased from CLEA Japan Inc. (Fujinomiya, Japan) and housed in a light-cycled room with controlled temperature and humidity.

For High-Performance Liquid Chromatography–Mass Spectrometry (LC-MS/MS) Reagent, Paclitaxel-d5 was purchased from Toronto Research Chemicals (North York, ON, Canada). Carboplatin-d4 was purchased from Cayman Chemical (Ann Arbor, MI, USA). Other solvents were obtained from FUJIFILM Wako Pure Chemical (Osaka, Japan).

### 2.3. Measurement of PTX and CBDCA Concentrations in Tumors with LC-MS/MS

**Figure 1:**
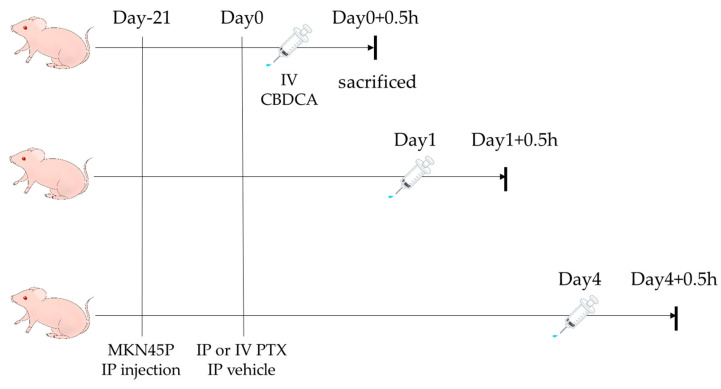
Experimental design to evaluate concentrations of PTX and CBDCA in peritoneal tumors. BALB/c nude mice were injected IP with 3 × 10⁶ MKN45P. After three weeks, PTX was administered IP or IV and vehicle was given IP. Right, 24 h and 96 h after PTX administration, CBDCA was injected IV and mice were sacrificed 30 min thereafter. Tumors were resected and PTX and CBDCA concentrations were measured with LC-MS/MS.

Five weeks after birth, mice were injected IP with MKN45P (3 × 10⁶) suspended in 500 µL Hanks’ Balanced Salt Solution (HBSS). After 3 weeks, when peritoneal tumors had formed, PTX was injected either IV via the tail vein or IP. Immediately after PTX administration, at 24 or 96 h, CBDCA was administered IV. Thirty min after CBDCA inoculation, mice were sacrificed, and tumors of similar size (over 1.0 mm in diameter) were resected and wiped with Kimwipes to remove PTX from tumor surfaces (Figure 1). Immediately after wiping, samples were placed in cryotubes, stored at −80 °C in liquid nitrogen, weighed, and homogenized to measure PTX and CBDCA concentrations (ng/mg) using LC-MS/MS (LCMS-8050 System, Shimadzu, Kyoto, Japan), as described below.

### 2.4. Preparation of Tumor Samples

Tumor samples were weighed (5–60 mg) into 2 mL hard tubes, to which 10 µL of internal standard reagents (10 µg/mL Paclitaxel-d5 and 10 µg/mL Carboplatin-d4) and 1 mL of acetonitrile had been added. Tumors were then homogenized and centrifuged (14,000 rpm for 10 min at 4 °C), and supernatants were analyzed.

### 2.5. Preparation of Calibration Standards and QC Samples

Stock calibration solutions of PTX and CBDCA were prepared at 1 mg/mL in MeOH and then stored at −30 °C. Standard samples (1–500 ng for PTX and 5–500 ng for CBDCA) and Quality Control (QC) samples (5–400 ng for PTX and CBDCA) were prepared. These MeOH solutions were added to empty test tubes followed by evaporation to dryness. A total of 30 µL of drug-free sample suspension (equivalent to 30 mg of tumor) were added to tubes containing 10 µL of internal standard reagents (10 µg/mL Paclitaxel-d5 and 10 µg/mL Carboplatin-d4) and 1 mL of acetonitrile. These were homogenized and centrifuged (14,000 rpm for 10 min at 4 °C), and supernatants were analyzed.

### 2.6. Analysis of PTX

The concentration of PTX was determined using a method we have previously described [28]. Briefly, for LC analyses, we utilized a YMC-Triart C_18_ analytical column (50 × 2 mm, 1.9 µm) with the column oven maintained at 40 °C and the autosampler set to 4 °C. The mobile phase consisted of 0.1% formic acid in water (Phase A) and acetonitrile (Phase B). The flow rate was 0.5 mL/min, and the injection volume was 3 μL. The gradient elution program was as follows: 40% B from 0 to 2.7 min; a linear increase to 100% B from 2.7 to 4 min; 100% B held from 4 to 6 min; 100% B to 40% B from 6 to 6.5 min; and a return to 40% B from 6.5 to 8 min

Detection of PTX and the internal standard PTX-d5 was performed in positive electrospray ionization (ESI) mode. Mass spectrometry/mass spectrometry (MS/MS) conditions were optimized as follows: nebulizer gas flow at 3 L/min, heating gas flow at 10 L/min, interface temperature at 300 °C, desolvation temperature at 526 °C, heat block temperature at 400 °C, and drying gas flow at 10 L/min. Collision energies were set at 22 V for PTX and 25 V for PTX-d5. Monitored mass transitions were *m*/*z* 854 > 286 for PTX and *m*/*z* 859 > 291 for PTX-d5.

### 2.7. Analysis of CBDCA

The concentration of CBDCA was analyzed using LC with an ACQUITY UPLC BEH HILIC analytical column (100 × 2.1 mm, 1.7 µm). The column oven was maintained at 40 °C, and the autosampler was set to 4 °C. The mobile phase consisted of 90% 10 mM ammonium formate in water and 10% acetonitrile (Phase A) and acetonitrile alone (Phase B). The flow rate was 0.3 mL/min, with an injection volume of 3 μL. The gradient elution program was as follows: 90% B from 0 to 2.5 min; a linear decrease to 30% B from 2.5 to 3 min; 30% B held from 3 to 4 min; and a return to 90% B from 4 to 7 min.

CBDCA and the internal standard Carboplatin-d4 were detected in positive ESI mode. The MS/MS conditions were as follows: nebulizer gas flow at 3 L/min, heating gas flow at 10 L/min, interface temperature at 200 °C, desolvation temperature at 355 °C, heat block temperature at 400 °C, and drying gas flow at 10 L/min. Collision energies were set at 19 V for CBDCA and 16 V for Carboplatin-d4. Monitored mass transitions were *m*/*z* 372 > 294 for CBDCA and *m*/*z* 376 > 298 for Carboplatin-d4.

Concentrations of PTX and CBDCA in tumor samples were quantified by comparing area ratios of analytes to those of internal standards (PTX-d5 and Carboplatin-d4) added to samples.

### 2.8. Ethics

All procedures were approved by the Animal Care Committee of Jichi Medical University (approval no. 23022-01) and performed in accordance with ARRIVE guidelines and Japanese Guidelines for Animal Research.

### 2.9. Statistical Analysis

Data are presented as means ± standard deviations. For data on concentrations of PTX and CBDCA, *p*-values were evaluated with the Mann–Whitney U test and one-way ANOVA, respectively. Statistical analysis was performed using Prism 9 (GraphPad Software, San Diego, CA, USA). Statistical significance was set at *p* < 0.05.

## 3. Results

### 3.1. Concentrations of PTX in Peritoneal Metastases of Gastric Cancer

**Figure 2:**
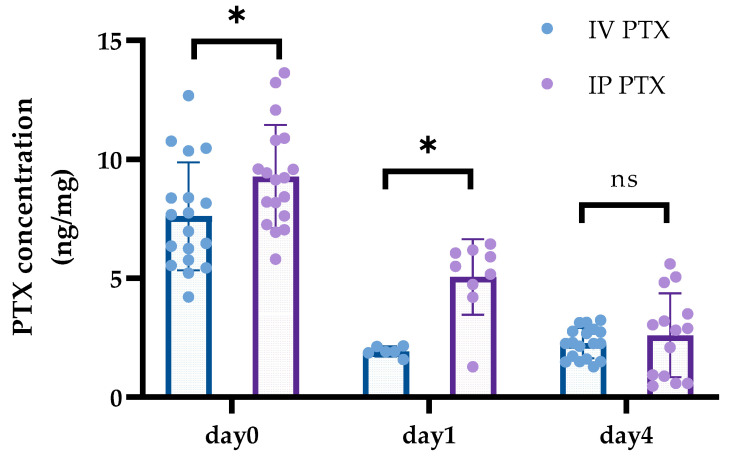
Concentrations of PTX in tumors after IV or IP PTX injection. The number of PMs from 14 to 18 was obtained from both groups at days 0 and 4 after PTX administration, and PTX concentrations were measured with LC-MS/MS. Both groups involved 3 or more mice. Day 1 groups comprised 6 to 9 samples. Both groups involved 1 or 2 mice. Data show the mean ± SD. *: *p* < 0.05, ns: not significant.

Concentrations of PTX, 0 and 1 day after PTX administration, were significantly higher in the IP group than the IV group (7.60 ± 2.27 ng/mg versus 9.27 ± 2.17 ng/mg, *p* = 0.020, 1.92 ± 0.20 ng/mg versus 5.05 ± 1.58 ng/mg, *p* = 0.011). However, at day 4, the concentration did not differ between the two groups (2.26 ± 0.63 ng/mg versus 2.60 ± 1.75 ng/mg, *p* = 0.45) (Figure 2). In the vehicle group, PTX was consistently undetectable in tumors.

### 3.2. Concentrations of CBDCA in Tumors after PTX Administration

**Figure 3:**
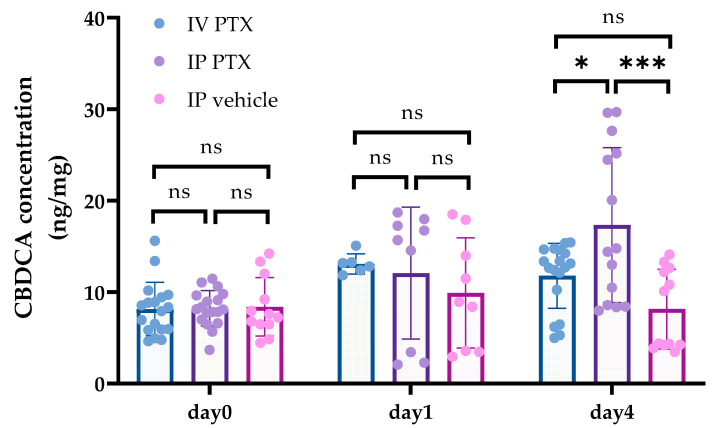
Concentrations of CBDCA in tumors after IV or IP PTX injection. Numbers of PMs from 12 to 18 were obtained at days 0 and 4, respectively, after PTX administration and CBDCA concentrations were measured. Each group involved 3 or more mice. Day 1 groups each comprised 1 or 2 mice. Data show the mean ± SD. *: *p* < 0.05, ***: *p* < 0.001, ns: not significant.

We measured CBDCA concentrations concurrently with PTX concentrations. There were no differences in CBDCA concentration between the IV and IP PTX groups at day 0 or day 1 after PTX administration (8.13 ± 2.92 ng/mg versus 8.22 ± 1.96 ng/mg, *p* = 0.99, 13.1 ± 1.1 ng/mg versus 12.08 ± 7.21 ng/mg, *p* = 0.94), whereas CBDCA concentrations 4 days after IP PTX were statistically higher in the IP group (11.78 ± 3.56 ng/mg versus 17.33 ± 8.47 ng/mg, *p* = 0.026) (Figure 3). Moreover, CBDCA concentrations after 4 days were higher than at day 0 in the IP PTX group (8.22 ± 1.96 ng/mg versus 17.33 ± 8.47 ng/mg, *p* = 0.0003).

### 3.3. Methods Validation of LC-MS/MS

**Figure 4:**
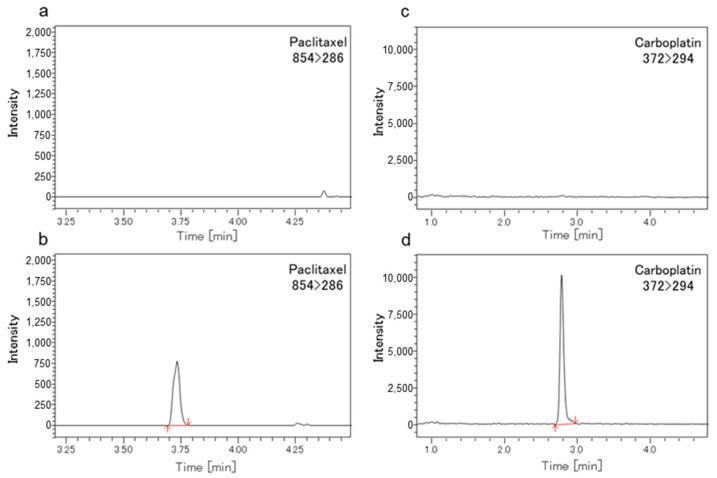
Chromatograms of Paclitaxel and Carboplatin spiked in mouse tumor samples. Representative chromatograms of (**a**) blank mouse tumor samples spiked with only paclitaxel-d5; (**b**) PTX (*m*/*z* 854 > 286; 3.7 min) added to blank mouse tumor samples at the LLOQ (lower limit of quantification) concentration of 1 ng; (**c**) blank mouse tumor samples spiked only with carboplatin-d4; and (**d**) CBDCA (*m*/*z* 372 > 294; 2.9 min) added to blank mouse tumor samples at the LLOQ concentration of 5 ng.

Figure 4 show chromatograms of mouse tumors spiked with PTX (Figure 4b) and CBDCA (Figure 4d). The column, mobile phase, and gradient program were optimized to obtain the best peak shape. Total run time was 6 min for PTX and 7 min for CBDCA with retention times of 3.7 min (PTX) and 2.9 min (CBDCA). Chromatograms in Figure 4a and Figure 4c show that no interfering substances were detected in blank samples.

## 4. Discussion

Although IP chemotherapy using taxane is effective for PMs, optimal treatment schedules remain undetermined, largely due to conflicting data. Consequently, our study focused on the timing between IP and IV administration to enhance the delivery of anti-cancer drugs to PMs. First, we demonstrated that PTX concentrations in PMs remained significantly higher in the IP PTX group compared to the IV group for at least 24 h post-administration. Previous studies have shown that high concentrations of PTX persist in the peritoneal cavity in humans after a single IP instillation, compared to IV administration [17,29]. However, there is limited information on PTX concentration in disseminated peritoneal tumors. Soma et al. demonstrated that drug concentrations in various internal organs (excluding tumors) remained consistently higher with IP administration than with IV administration for 24 h in rabbits [30]. Moreover, the same group reported that PTX concentrations in PMs in mice, measured over 24 h, gradually decreased over time [31]. However, no study has examined PTX concentrations in PMs beyond 24 h. Interestingly, although values in the IP group remained close to those in the IV group at day 4, the results of our study mostly align with these findings, indicating that IP administration is a suitable route for delivering PTX to PMs because IP PTX is superior to IV PTX against PMs in terms of drug concentrations at day 1 after PTX administration and not inferior to at least day 4.

A more significant finding in this study is that IP PTX enhances the drug delivery efficiency of systemically administered CBDCA to PMs. Joshi et al. reported that IV CBDCA concentrations in mouse kidney and bone marrow gradually decreased until 6 h post-injection [32], and Jarvis et al. demonstrated that IP CBDCA concentrations in ovarian tumors peaked 30 min post-injection in a mouse model [33]. Considering these previous studies, we examined CBDCA concentrations in peritoneal tumors 30 min after CBDCA administration. We observed that CBDCA concentrations increased when administered 4 days after IP PTX, with values significantly higher than those following IV PTX at the same interval. Conversely, CBDCA levels showed minimal difference between IP and IV PTX administration at days 0 and 1. These findings suggest that the interval between IP and IV administration is crucial for enhancing drug concentration in tumors and the efficacy of IV-administered drugs on PMs.

**Figure 5:**
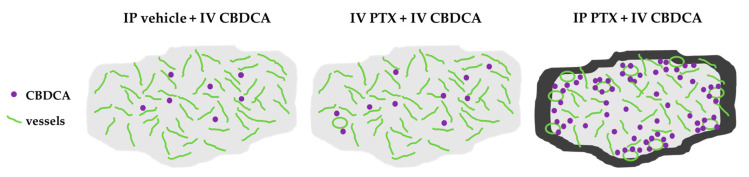
Schematic illustration of a hypothesis for increasing IV CBDCA concentrations in tumors 4 days after IP PTX injection. The bold black line in the tumor margin indicates apoptosis induced by IP PTX. Microvessels open and reduce interstitial pressure in PMs, improving drug delivery at day 4 because of apoptosis of peripheral tumor cells.

The exact mechanism underlying this phenomenon was not identified; however, we hypothesize that tumor vessels may become more permeable, allowing drugs to spread more easily in peritoneal tumors after IP PTX. This hypothesis is based on the observation that IP PTX directly penetrates PMs from both sides of the tumor surface, inducing massive apoptosis of peripheral tumor cells [16]. Moreover, IP PTX destroys peripheral microvessels in tumors, significantly reducing the number of CD31-positive vessel structures superficially, but not at the tumor center [34]. The overall proportion of vessels or the number of open vessels was not evaluated in this study, but our research supported the hypothesis that IP PTX decreases interstitial pressure, alters vascular occlusion, and restores blood flow, thereby enhancing the delivery of anti-cancer drugs to PMs (Figure 5). If our hypothesis is correct, this approach could improve drug delivery and reduce hypoxia in the tumor microenvironment (TME), thereby increasing drug efficacy and reducing drug resistance by preventing changes in drug metabolism [35].

Although hyperthermic intraperitoneal chemotherapy (HIPEC) has demonstrated some efficacy in treating gastric cancer [36,37,38], it is not currently considered a replacement for systemic chemotherapy. While HIPEC may prolong survival by modifying the type, temperature, and retention time of anti-cancer drugs, it is generally administered only once, immediately following cytoreductive surgery. However, since the infiltration distance of IP-administered PTX is limited, systemic chemotherapy is essential to maximize the therapeutic impact on PMs. Kamei et al. reported that conventional PTX solubilized with Cremophor EL localized only 100–200 µm from the surface of PMs in fluorescence-labeled gastric cancer mouse models [16]. The significant difference in CBDCA concentrations between the IV and IP PTX groups, nearly two-fold when administered 4 days after IP PTX, substantially enhances the anti-tumor effects of IV-administered drugs against PMs.

IP chemotherapy can be given repeatedly via a subcutaneous port connected to an intraperitoneal catheter, avoiding the need for additional surgery. Multiple cycles of IP PTX and IV chemotherapy, administered at appropriate time intervals, can create a positive feedback loop and potentially enhance anti-tumor effects against PMs. Therefore, a repeated treatment combining IP PTX and systemic chemotherapy in a bidirectional manner represents an ideal strategy for treating PMs.

In summary, we have determined the optimal timing between IP PTX and subsequent systemic chemotherapy to enhance the pharmacokinetics of IV-administered drugs. With further improvements, this strategy may be applicable not only to gastric cancer but also to various other types of tumors, such as ovarian and colorectal cancers [39], and could establish a potential role for combined IP and systemic chemotherapy. More studies are needed to determine the most suitable IP and IV drugs as well as the optimal timing for their administration and to evaluate tumor responses to anti-cancer drugs and survival times of animals repeatedly for clinical application, which may lead to improved prognoses for patients with PMs.

## 5. Conclusions

Using a mouse model, we found that sequential systemic chemotherapy following IP PTX increased the concentration of IV-administered drugs in PMs more effectively than simultaneous administration. This approach may enhance anti-cancer effects against PMs and improve prognoses for patients with this challenging condition.

## Data Availability

The data presented in this study are available on request from the corresponding author.

## References

[1] Sung H., Ferlay J., Siegel R.L., Laversanne M., Soerjomataram I., Jemal A., Bray F. (2021). Global Cancer Statistics 2020: GLOBOCAN Estimates of Incidence and Mortality Worldwide for 36 Cancers in 185 Countries. CA Cancer J. Clin..

[2] Yarema R., Ohorchak M., Hyrya P., Kovalchuk Y., Safiyan V., Karelin I., Ferneza S., Fetsych M., Matusyak M., Oliynyk Y. (2020). Gastric cancer with peritoneal metastases: Efficiency of standard treatment methods. World J. Gastrointest. Oncol..

[3] Kawabata R., Fujitani K., Sakamaki K., Ando M., Ito Y., Tanizawa Y., Yamada T., Hirao M., Yamada M., Hihara J. (2022). Survival analysis of a prospective multicenter observational study on surgical palliation among patients with malignant bowel obstruction caused by peritoneal dissemination of gastric cancer. Gastric Cancer.

[4] Yoo C.H., Noh S.H., Shin D.W., Choi S.H., Min J.S. (2000). Recurrence following curative resection for gastric carcinoma. Br. J. Surg..

[5] Roviello F., Marrelli D., de Manzoni G., Morgagni P., Di Leo A., Saragoni L., De Stefano A. (2003). Prospective study of peritoneal recurrence after curative surgery for gastric cancer. Br. J. Surg..

[6] Bilici A., Selcukbiricik F. (2015). Prognostic significance of the recurrence pattern and risk factors for recurrence in patients with proximal gastric cancer who underwent curative gastrectomy. Tumour Biol..

[7] Koizumi W., Narahara H., Hara T., Takagane A., Akiya T., Takagi M., Miyashita K., Nishizaki T., Kobayashi O., Takiyama W. (2008). S-1 plus cisplatin versus S-1 alone for first-line treatment of advanced gastric cancer (SPIRITS trial): A phase III trial. Lancet Oncol..

[8] Kang Y.K., Kang W.K., Shin D.B., Chen J., Xiong J., Wang J., Lichinitser M., Guan Z., Khasanov R., Zheng L. (2009). Capecitabine/cisplatin versus 5-fluorouracil/cisplatin as first-line therapy in patients with advanced gastric cancer: A randomised phase III noninferiority trial. Ann. Oncol..

[9] Fujitani K., Yang H.K., Mizusawa J., Kim Y.W., Terashima M., Han S.U., Iwasaki Y., Hyung W.J., Takagane A., Park D.J. (2016). Gastrectomy plus chemotherapy versus chemotherapy alone for advanced gastric cancer with a single non-curable factor (REGATTA): A phase 3, randomised controlled trial. Lancet Oncol..

[10] Shirao K., Boku N., Yamada Y., Yamaguchi K., Doi T., Goto M., Nasu J., Denda T., Hamamoto Y., Takashima A. (2013). Randomized Phase III study of 5-fluorouracil continuous infusion vs. sequential methotrexate and 5-fluorouracil therapy in far advanced gastric cancer with peritoneal metastasis (JCOG0106). Jpn. J. Clin. Oncol..

[11] Nakajima T.K., Yamaguchi K., Boku N., Hyodo I., Mizusawa J., Hara H., Nishina T., Sakamoto T., Shitara K., Shinozaki K. (2020). Randomized phase II/III study of 5-fluorouracil/l-leucovorin versus 5-fluorouracil/l-leucovorin plus paclitaxel administered to patients with severe peritoneal metastases of gastric cancer (JCOG1108/WJOG7312G). Gastric Cancer..

[12] Jacquet P., Sugarbaker P.H. (1996). Peritoneal-plasma barrier. Cancer Treat Res..

[13] Dedrick R.L., Myers C.E., Bungay P.M., DeVita V.T. (1978). Pharmacokinetic rationale for peritoneal drug administration in the treatment of ovarian cancer. Cancer Treat Rep..

[14] Los G., Mutsaers P.H., van der Vijgh W.J., Baldew G.S., de Graaf P.W., McVie J.G. (1989). Direct diffusion of cis-diamminedichloroplatinum(II) in intraperitoneal rat tumors after intraperitoneal chemotherapy: A comparison with systemic chemotherapy. Cancer Res..

[15] Flessner M.F., Fenstermacher J.D., Blasberg R.G., Dedrick R.L. (1985). Peritoneal absorption of macromolecules studied by quantitative autoradiography. Am. J. Physiol..

[16] Kamei T., Kitayama J., Yamaguchi H., Soma D., Emoto S., Konno T., Ishihara K., Ishigami H., Kaisaki S., Nagawa H. (2011). Spatial distribution of intraperitoneally administrated paclitaxel nanoparticles solubilized with poly (2-methacryloxyethyl phosphorylcholine-co n-butyl methacrylate) in peritoneal metastatic nodules. Cancer Sci..

[17] Markman M., Rowinsky E., Hakes T., Reichman B., Jones W., Lewis J.L., Rubin S., Curtin J., Barakat R., Phillips M. (1992). Phase I trial of intraperitoneal taxol: A Gynecoloic Oncology Group study. J. Clin. Oncol..

[18] Armstrong D.K., Bundy B., Wenzel L., Huang H.Q., Baergen R., Lele S., Copeland L.J., Walker J.L., Burger R.A. (2006). Intraperitoneal cisplatin and paclitaxel in ovarian cancer. N. Engl. J. Med..

[19] Rufián S., Muñoz-Casares F.C., Briceño J., Díaz C.J., Rubio M.J., Ortega R., Ciria R., Morillo M., Aranda E., Muntané J. (2006). Radical surgery-peritonectomy and intraoperative intraperitoneal chemotherapy for the treatment of peritoneal carcinomatosis in recurrent or primary ovarian cancer. J. Surg. Oncol..

[20] Yang S., Feng R., Pan Z.C., Jiang T., Xu Q., Chen Q. (2015). A Comparison of Intravenous plus Intraperitoneal Chemotherapy with Intravenous Chemotherapy Alone for the Treatment of Gastric Cancer: A Meta-Analysis. Sci. Rep..

[21] Ishigami H., Fujiwara Y., Fukushima R., Nashimoto A., Yabusaki H., Imano M., Imamoto H., Kodera Y., Uenosono Y., Amagai K. (2018). Phase III Trial Comparing Intraperitoneal and Intravenous Paclitaxel Plus S-1 Versus Cisplatin Plus S-1 in Patients With Gastric Cancer With Peritoneal Metastasis: PHOENIX-GC Trial. J. Clin. Oncol..

[22] Hosie K., Gilbert J.A., Kerr D., Brown C.B., Peers E.M. (2001). Fluid dynamics in man of an intraperitoneal drug delivery solution: 4% icodextrin. Drug Deliv..

[23] Mohamed F., Marchettini P., Stuart O.A., Sugarbaker P.H. (2003). Pharmacokinetics and tissue distribution of intraperitoneal paclitaxel with different carrier solutions. Cancer Chemother. Pharmacol..

[24] Bregoli L., Movia D., Gavigan-Imedio J.D., Lysaght J., Reynolds J., Prina-Mello A. (2016). Nanomedicine applied to translational oncology: A future perspective on cancer treatment. Nanomedicine.

[25] Yamashita K., Tsunoda S., Gunji S., Murakami T., Suzuki T., Tabata Y., Sakai Y. (2019). Intraperitoneal chemotherapy for peritoneal metastases using sustained release formula of cisplatin-incorporated gelatin hydrogel granules. Surg. Today.

[26] Nagao S., Fujiwara K., Yamamoto K., Tanabe H., Okamoto A., Takehara K., Saito M., Fujiwara H., Tan D.S.P., Yamaguchi S. (2023). Intraperitoneal Carboplatin for Ovarian Cancer—A Phase 2/3 Trial. NEJM Evid..

[27] Sako A., Kitayama J., Koyama H., Ueno H., Uchida H., Hamada H., Nagawa H. (2004). Transduction of soluble Flt-1 gene to peritoneal mesothelial cells can effectively suppress peritoneal metastasis of gastric cancer. Cancer Res..

[28] Saito A., Kimura N., Kaneda Y., Ohzawa H., Miyato H., Yamaguchi H., Lefor A.K., Nagai R., Sata N., Kitayama J. (2022). Novel Drug Delivery Method Targeting Para-Aortic Lymph Nodes by Retrograde Infusion of Paclitaxel into Pigs’ Thoracic Duct. Cancers.

[29] Francis P., Rowinsky E., Schneider J., Hakes T., Hoskins W., Markman M. (1995). Phase I feasibility and pharmacologic study of weekly intraperitoneal paclitaxel: A Gynecologic Oncology Group pilot Study. J. Clin. Oncol..

[30] Soma D., Kitayama J., Ishigami H., Kaisaki S., Nagawa H. (2009). Different tissue distribution of paclitaxel with intravenous and intraperitoneal administration. J. Surg. Res..

[31] Emoto S., Yamaguchi H., Kamei T., Ishigami H., Suhara T., Suzuki Y., Ito T., Kitayama J., Watanabe T. (2014). Intraperitoneal administration of cisplatin via an in situ cross-linkable hyaluronic acid-based hydrogel for peritoneal dissemination of gastric cancer. Surg. Today.

[32] Joshi A., Guo J., Holleran J.L., Kiesel B., Taylor S., Christner S., Parise R.A., Miller B.M., Ivy S.P., Chu E. (2020). Evaluation of the pharmacokinetic drug-drug interaction potential of iohexol, a renal filtration marker. Cancer Chemother. Pharmacol..

[33] Jarvis I.W.H., Meczes E.L., Thomas H.D., Edmondson R.J., Veal G.J., Boddy A.V., Ottley C.J., Pearson D.G., Tilby M.J. (2012). Therapy-induced carboplatin-DNA adduct levels in human ovarian tumours in relation to assessment of adduct measurement in mouse tissues. Biochem. Pharmacol..

[34] Kitayama J., Emoto S., Yamaguchi H., Ishigami H., Watanabe T. (2014). Intraperitoneal paclitaxel induces regression of peritoneal metastasis partly by destruction of peripheral microvessels. Cancer Chemother. Pharmacol..

[35] El-Tanani M., Rabbani S.A., Babiker R., Rangraze I., Kapre S., Palakurthi S.S., Alnuqaydan A.M., Aljabali A.A., Rizzo M., El-Tanani Y. (2024). Unraveling the tumor microenvironment: Insights into cancer metastasis and therapeutic strategies. Cancer Lett..

[36] Glehen O., Gilly F.N., Arvieux C., Cotte E., Boutitie F., Mansvelt B., Bereder J.M., Lorimier G., Quenet F., Elias D. (2010). Peritoneal carcinomatosis from gastric cancer: A multi-institutional study of 159 patients treated by cytoreductive surgery combined with perioperative intraperitoneal chemotherapy. Ann. Surg. Oncol..

[37] Rudloff U., Langan R.C., Mullinax J.E., Beane J.D., Steinberg S.M., Beresnev T., Webb C.C., Walker M., Toomey M.A., Schrump D. (2014). Impact of maximal cytoreductive surgery plus regional heated intraperitoneal chemotherapy (HIPEC) on outcome of patients with peritoneal carcinomatosis of gastric origin: Results of the GYMSSA trial. J. Surg. Oncol..

[38] Yonemura Y., Kawamura T., Bandou E., Takahashi S., Sawa T., Matsuki N. (2005). Treatment of peritoneal dissemination from gastric cancer by peritonectomy and chemohyperthermic peritoneal perfusion. Br. J. Surg..

[39] Murono K., Nozawa H., Nagata H., Ishimaru K., Sonoda H., Emoto S., Kaneko M., Sasaki K., Otani K., Kawai K. (2020). Efficacy of intraperitoneally administered paclitaxel for colorectal cancer with peritoneal metastases. Int. J. Color. Dis..

